# Anatomical Variation of the Posterior Tibial Neurovascular Bundle During Posterior Ankle Arthroscopy for Posterior Ankle Impingement Syndrome: A Case Report

**DOI:** 10.7759/cureus.112251

**Published:** 2026-07-08

**Authors:** Robert J Rigby, Christopher J Pearce

**Affiliations:** 1 College of Medicine and Health, University of Birmingham, Birmingham, GBR; 2 Foot and Ankle Surgery, Altius Clinic, Singapore, SGP

**Keywords:** anatomical variation, posterior ankle arthroscopy, posterior ankle impingement, posterior tibial neurovascular bundle, tibial nerve injury

## Abstract

We report the case of a 32-year-old male professional soccer player presenting with a one-year history of posterior ankle pain due to posterior ankle impingement syndrome secondary to an elongated Stieda process and loose bodies. Preoperative review of MRI revealed an anatomical variation of the posterior neurovascular bundle in relation to the flexor hallucis longus (FHL) tendon at the level of the ankle joint. In this case, the neurovascular bundle was situated posterior and lateral to the FHL tendon, as opposed to medial to the FHL tendon, as seen in typical anatomy. This finding is significant because posterior ankle arthroscopy relies on the FHL tendon as an anatomical landmark to protect the posterior neurovascular bundle by always working lateral and posterior to the tendon. An arthroscopic approach would have posed a high risk of iatrogenic injury to the tibial nerve and posterior tibial vessels. The originally planned arthroscopic ankle debridement was therefore changed to an open approach using a posteromedial incision. Postoperative recovery was uncomplicated, and the patient returned to soccer within seven weeks with little swelling and no pain.

## Introduction

Posterior ankle impingement syndrome (PAIS) is a cause of chronic posterior ankle pain, commonly affecting athletes in sports involving repetitive plantar flexion. It often results from osseous abnormalities such as an os trigonum (an accessory ossicle posterior to the talus), an elongated Stieda process (a prominent lateral tubercle of the posterior talar process), loose bodies, or may be due to purely soft tissue impingement [1]. PAIS is identified across a range of sports, including ballet, soccer, and high jump [2,3].

Posterior ankle arthroscopy is a well-known minimally invasive surgery for PAIS, relying on the identification of key anatomical landmarks to guide the surgeon. This arthroscopic technique is performed through posterolateral and posteromedial portals located adjacent to the lateral and medial borders of the Achilles tendon at the level of the lateral malleolus [2]. The posterior tibial neurovascular bundle normally lies immediately medial to the flexor hallucis longus (FHL) tendon within the tarsal tunnel [2,4]. Following portal placement, the FHL tendon is located, as it serves as the key intraoperative landmark, forming the medial border of the arthroscopic working area [2,4]. Instrumentation is kept lateral to the FHL tendon to reduce the risk of injury to the tibial nerve and posterior tibial vessels [2,4]. However, anatomical variations can significantly alter this relationship, increasing the risk of neurovascular injury if not identified.

This case report describes an unusual anatomical variation in which the posterior tibial neurovascular bundle was found in a posterolateral position relative to the FHL, identified preoperatively on magnetic resonance imaging (MRI). To ensure neurovascular protection, an open approach was adopted instead of the previously planned arthroscopic technique. Intraoperative findings confirmed the abnormal anatomy. To our knowledge, no previous reports describe a posterolateral posterior tibial neurovascular bundle relative to the FHL tendon in the setting of posterior ankle arthroscopy, adding to its unique nature. This highlights the importance of preoperative imaging review and flexibility in surgical planning.

## Case presentation

A 32-year-old professional soccer player at the national team level presented in February 2026 with a one-year history of progressive posterior ankle pain. Three weeks prior to presentation, he experienced an acute exacerbation during a competitive match, describing a sharp onset of posterior ankle pain. Following this episode, symptoms worsened significantly and became more diffuse throughout the ankle.

A corticosteroid injection administered 10 days prior to review resulted in partial symptom relief, allowing progression from walking to light jogging and improved subjective stability. However, the patient continued to report a “pinching” sensation at specific angles of plantar flexion.

There was no visible swelling. Hindfoot alignment demonstrated mild varus. The ankle exhibited a normal range of motion. Palpation revealed anteromedial tenderness due to spurs. Both anterior impingement and posterior impingement tests were positive. Subtalar joint motion, including inversion and eversion, was slightly restricted. The ankle was stable on ligamentous assessment.

**Figure 1:**
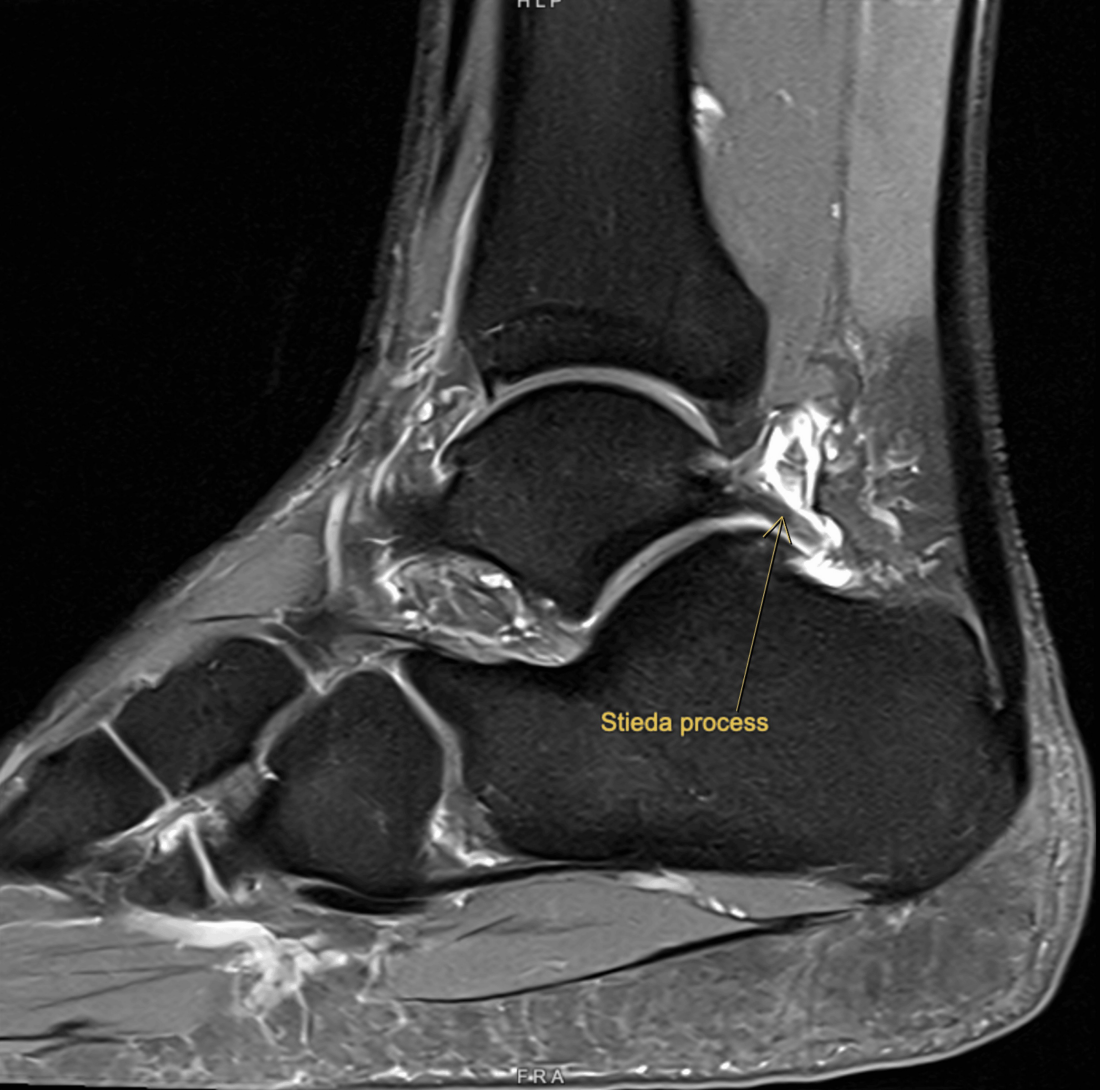
Sagittal T2-weighted MRI of the left ankle Sagittal fat-suppressed T2-weighted MRI of the left ankle demonstrating features of posterior ankle impingement, including an elongated Stieda process with associated bone marrow edema.

**Figure 2:**
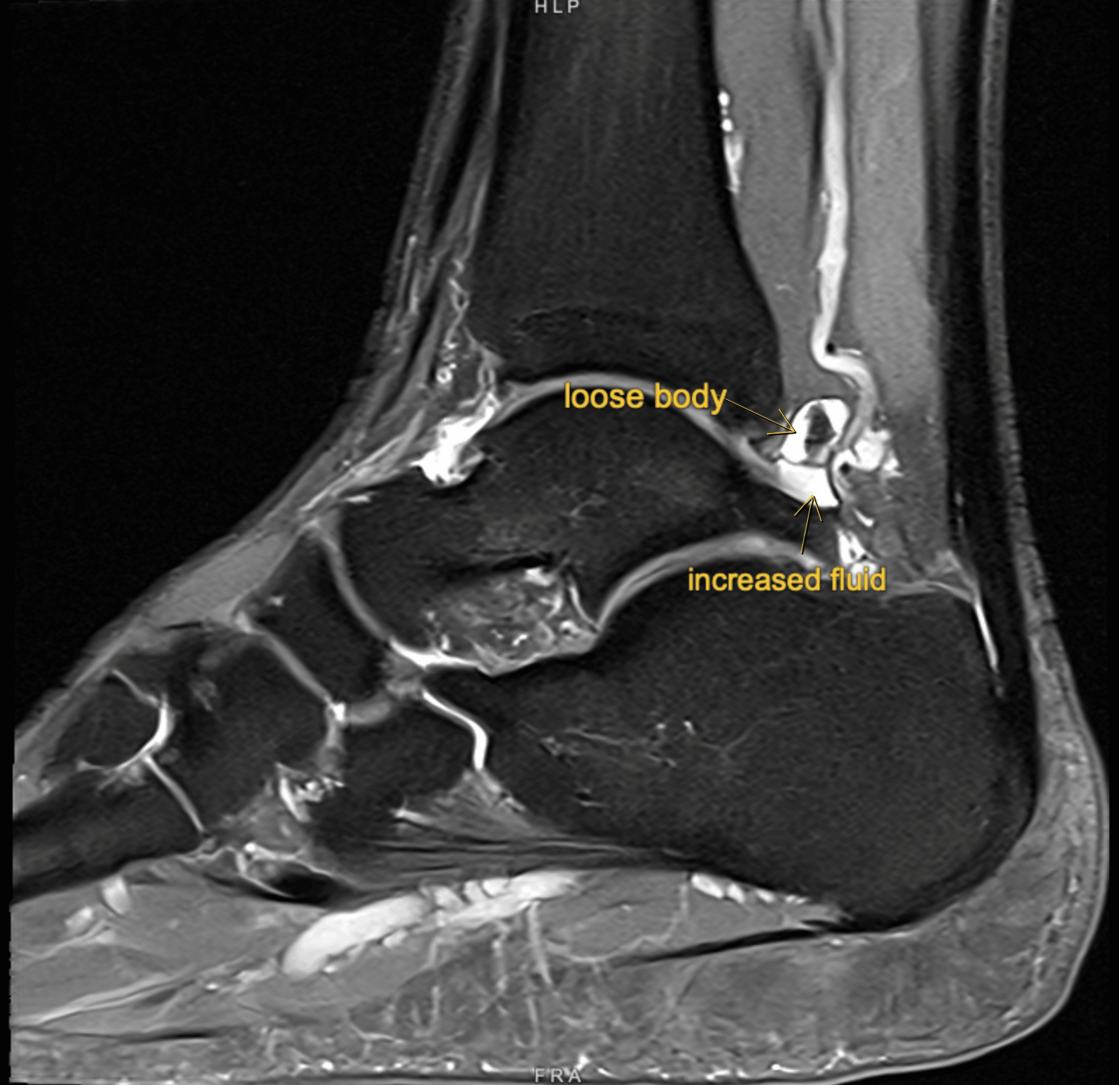
Sagittal MRI of the left ankle demonstrating posterior ankle loose body and increased joint fluid Sagittal fat-suppressed T2-weighted MRI demonstrating a posterior intra-articular loose body (arrow) with associated increased joint fluid, consistent with posterior ankle impingement.

An MRI scan from April 2025, obtained during a previous referral, demonstrated anterior and posterior ankle impingement, with a prominent medial spur and an elongated os trigonum (Figure 1). There was associated bone marrow edema, posterior joint fluid, and multiple loose fragments (Figure 2). Ligamentous appearances were consistent with prior repair. 

Diagnosis was combined anterior and posterior ankle impingement with multiple loose bodies and an elongated Stieda process, associated with bone marrow edema. The plan on Day 1 was to use arthroscopic debridement to address the posterior impingement by excision of the elongated Stieda process and loose fragments.

**Figure 3:**
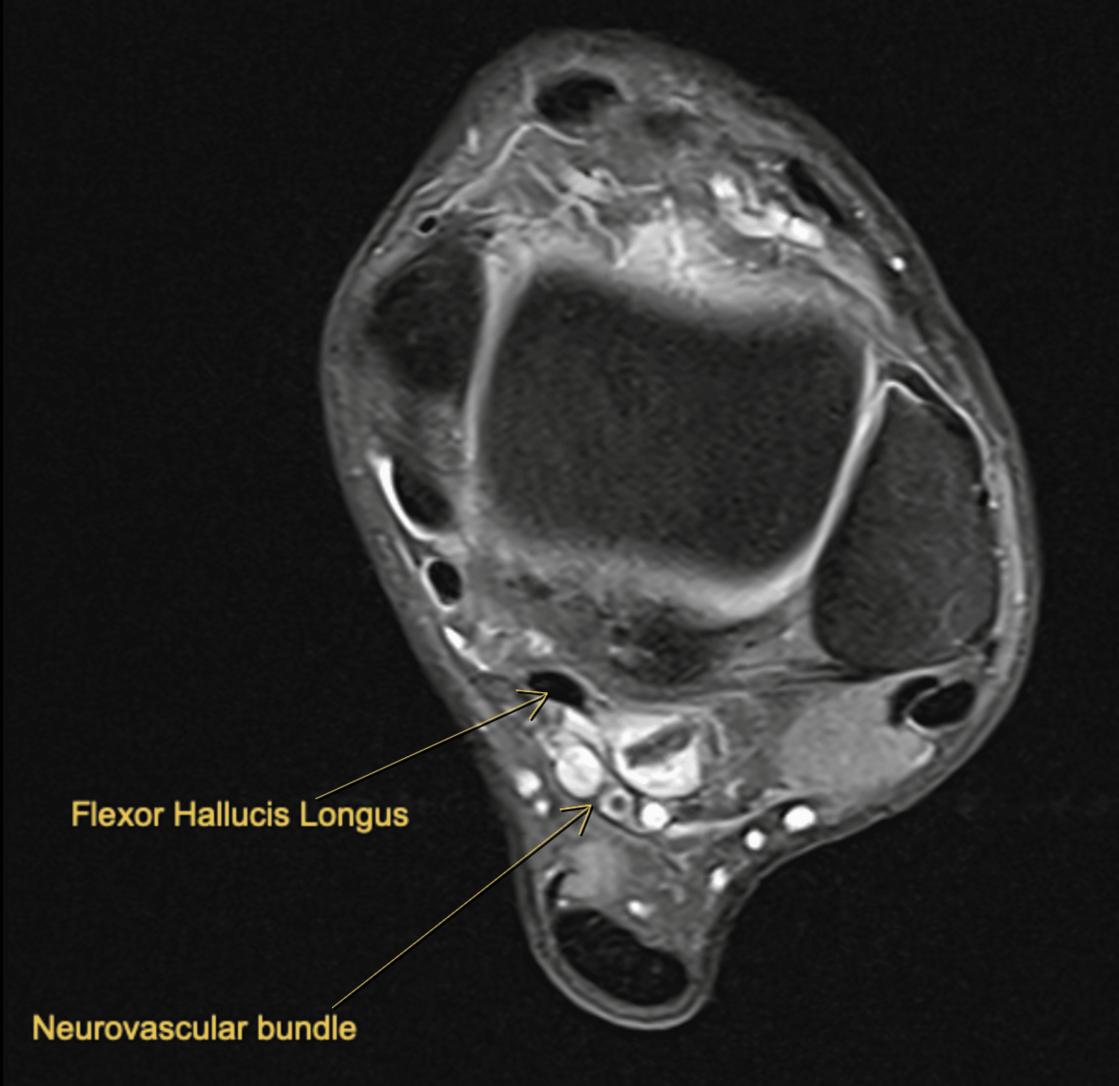
Axial fat-suppressed T2-weighted MRI of the left ankle Axial fat-suppressed T2-weighted MRI of the left ankle demonstrating the relationship between the flexor hallucis longus (FHL) tendon and the posterior tibial neurovascular bundle. The neurovascular bundle is located posterolateral to the FHL tendon, representing an atypical anatomical configuration.

On Day 2, the MRI was viewed in more detail for surgical planning, and the atypical posterolateral position of the posterior tibial neurovascular bundle relative to the flexor hallucis longus was noticed. In normal anatomy, the posterior tibial neurovascular bundle is located immediately medial to the FHL tendon, but in this case, the neurovascular bundle was found posterolaterally, on the completely opposite side of the FHL tendon. The scans were reviewed with the patient and physiotherapist (Figure 3). Given the increased risk of tibial nerve injury with a standard posterior arthroscopic approach, a posteromedial open approach was selected to allow direct identification and protection of the neurovascular bundle. The case was also discussed with international foot and ankle sports surgeon colleagues, none of whom had ever come across this anatomical variant before. Postoperatively, recovery was uncomplicated. At the one-week review, wounds were healing well with no signs of infection, and neurovascular status was intact. Mobilization with physiotherapy and weight-bearing as tolerated was commenced. At 12 days, wounds were clean and dry, and swelling was minimal. The sutures were removed, and physiotherapy was ramped up. At seven weeks, the patient was pain-free with full range of ankle motion and negative impingement tests. He had returned to soccer without limitation, with only mild residual posterior incision swelling noted. 

## Discussion

Posterior ankle arthroscopy, a well-established procedure for the treatment of posterior ankle impingement syndrome, involves a two-portal technique described by van Dijk et al. [4]. A key principle of this approach is the use of the FHL tendon as an anatomical landmark to protect the posterior tibial neurovascular bundle [2,4].

The posterior tibial neurovascular bundle is typically located immediately medial to the FHL tendon within the deep posterior compartment of the ankle [5]. This consistent anatomical relationship underpins the safe zone described in posterior ankle arthroscopy, with surgical instruments directed laterally to reduce the risk of neurovascular injury [2].

**Figure 4:**
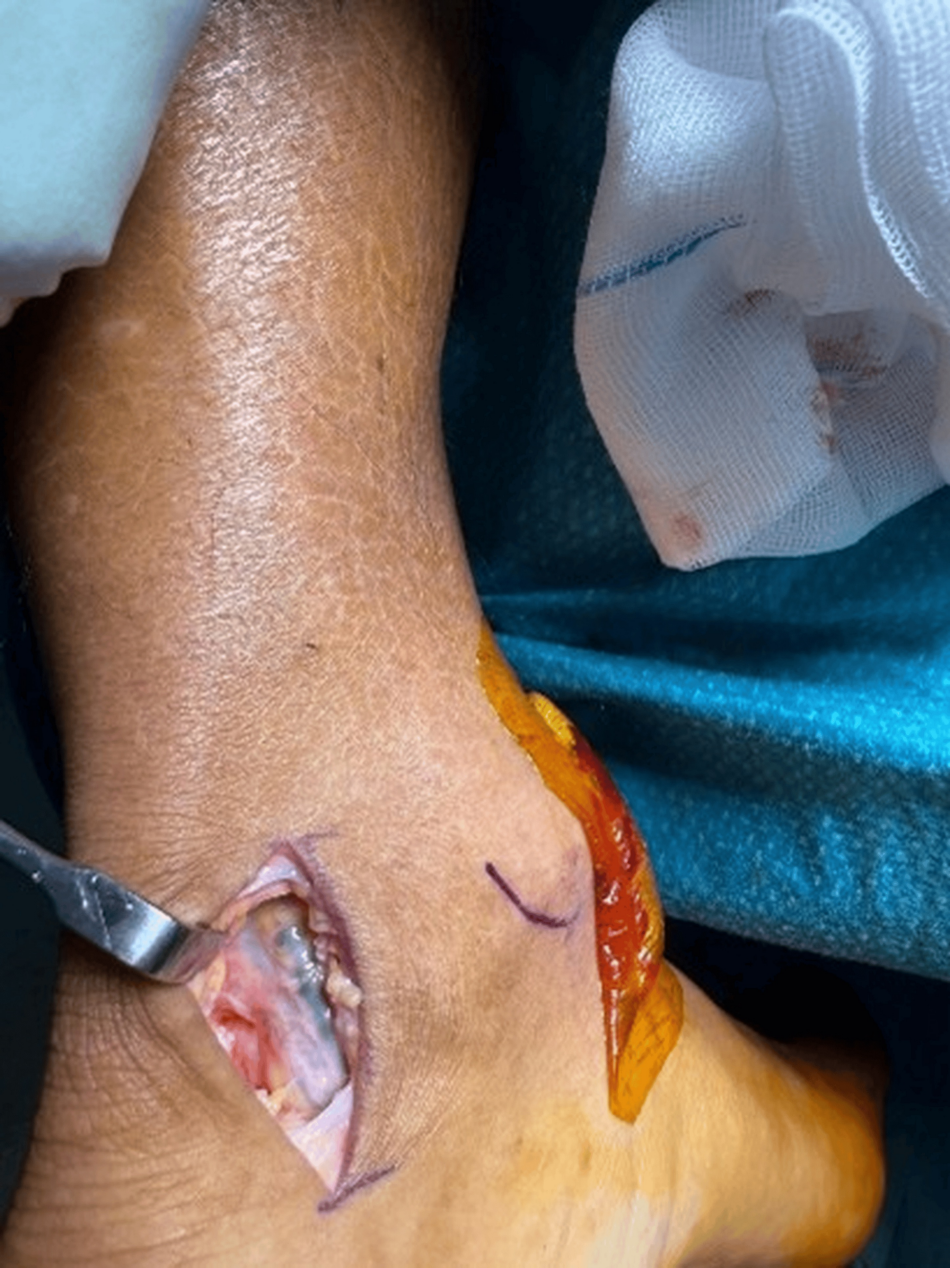
Intraoperative posteromedial view of the left posterior ankle compartment Original image of Intraoperative posteromedial view of left posterior ankle compartment demonstrating neurovascular bundle located posterior and lateral to the flexor hallucis longus tendon.

In the present case, the posterior tibial neurovascular bundle was in an atypical posterolateral position relative to the FHL tendon (Figure 4), representing a deviation from the expected anatomy [4]. Had the standard arthroscopic approach been used, the risk of iatrogenic neurovascular bundle injury would have been high [6]. Injury to the tibial nerve can result in plantar neuropathic pain, sensory loss, and intrinsic muscle atrophy [6]. The artery and vein would also have been at risk. 

This anatomical variation directly influenced surgical decision-making. Preoperative review of the MRI revealed the abnormal neurovascular bundle position, prompting the change from an arthroscopic approach to an open approach. A posteromedial open approach was selected to allow direct visualization and protection of the neurovascular bundle, reducing the risk of iatrogenic injury.

To our knowledge, this posterolateral neurovascular position has not been previously described. A literature search was carried out to identify previously reported cases or studies. PubMed and Embase databases were searched from inception to July 2026. The search strategy involved combinations of keywords and Medical Subject Headings (MeSH), including "posterior ankle arthroscopy," "posterior ankle impingement," "flexor hallucis longus," and "posterior tibial neurovascular bundle," alongside terms related to anatomical variation. The reference lists of relevant articles were reviewed to screen for additional studies.

This case highlights the importance of recognizing anatomical variance and carefully reviewing preoperative imaging before posterior ankle arthroscopy. The FHL tendon remains a useful anatomical landmark, but surgeons should be aware that anatomical variation can alter these relationships and compromise its reliability. Recognition of variants and flexibility in surgical planning are needed to ensure surgical safety and optimal patient outcomes.

## Conclusions

This case highlights the clinical significance of anatomical variance in the posterior tibial neurovascular bundle during posterior ankle arthroscopy. The flexor hallucis longus tendon is commonly considered to be a reliable anatomical landmark, with the neurovascular bundle located on its medial border, for safe portal placement. Deviations in the expected anatomy, such as the posterolateral location in this case, can increase the risk of iatrogenic injury. Preoperative imaging review, intraoperative assessment, and flexibility in the surgical approach are essential to provide neurovascular protection. Awareness of these anatomical variants can help surgeons improve safety and outcomes in the management of posterior ankle impingement syndrome.
